# Antigen-specific memory and naïve CD4^+^ T cells following secondary *Chlamydia trachomatis* infection

**DOI:** 10.1371/journal.pone.0240670

**Published:** 2020-10-22

**Authors:** Jennifer D. Helble, Alexander O. Mann, Michael N. Starnbach

**Affiliations:** 1 Department of Microbiology, Harvard Medical School, Boston, MA, United States of America; 2 Department of Immunology, Harvard Medical School, Boston, MA, United States of America; CCAC, UNITED STATES

## Abstract

Memory antigen-specific CD4^+^ T cells against *Chlamydia trachomatis* are necessary for protection against secondary genital tract infection. While it is known that naïve antigen-specific CD4^+^ T cells can traffic to the genital tract in an antigen-specific manner, these T cells are not protective during primary infection. Here, we sought to compare the differences between memory and naïve antigen-specific CD4^+^ T cells in the same mouse following secondary infection using transgenic CD4^+^ T cells (NR1 T cells). Using RNA sequencing, we found that there were subtle but distinct differences between these two T cell populations. Naïve NR1 T cells significantly upregulated cell cycle genes and were more proliferative than memory NR1 T cells in the draining lymph node. In contrast, memory NR1 T cells were more activated than naïve NR1 T cells and were enriched in the genital tract. Together, our data provide insight into the differences between memory and naïve antigen-specific CD4^+^ T cells during *C*. *trachomatis* infection.

## Introduction

During primary infection, professional antigen presenting cells process microbial antigens and present the resulting peptides in the context of MHC II. Circulating naïve CD4^+^ T cells with T cell receptor (TCR) specificity for these peptide: MHC II complexes bind, become activated, proliferate, and if necessary home to peripheral tissues to secrete cytokines and assist in pathogen clearance [1–5]. Such CD4^+^ T cells are critical components of the immune response against the bacterium *Chlamydia trachomatis*, the leading bacterial sexually transmitted infection in the United States [6–8]. During *C*. *trachomatis* infection in mice, CD4^+^ T cells are polarized as Th1-like and secrete high levels of the cytokine interferon-γ (IFNγ) [9, 10]. Following pathogen clearance in mice, antigen-specific CD4^+^ T cells can form a stable memory population that are reactivated during secondary infection [2, 6, 11]. While CD4^+^ T cell polarization and formation of a memory population is less clear during human *C*. *trachomatis* infection, there is evidence to suggest that both Th1 and Th2 populations are present and may protect against subsequent infections [12–16].

We have been able to study antigen-specific CD4^+^ T cells through the use of TCR transgenic cells specific for the *C*. *trachomatis* protein Cta1 (*Chlamydia* T cell antigen 1), denoted NR1 T cells [17]. The ability of antigen-specific CD4^+^ T cells to clear *C*. *trachomatis* infection is directly dependent on T cell trafficking to the genital tract, as mice that receive NR1 T cells that are defective in their ability to traffic to the genital tract exhibit higher bacterial burden than mice that receive functional NR1 T cells [18, 19]. Using these reagents, we have previously uncovered aspects of the antigen-specific CD4^+^ T cell response that generate protective immunity during primary infection, including chemokine receptors required for T cell homing to the tissue and host sensing of IFNγ [6, 9, 20].

Furthermore, it has been well established that memory antigen-specific CD4^+^ T cells are able to clear *C*. *trachomatis* infection during secondary infection [6, 9]. However, the possible contribution of naïve antigen-specific CD4^+^ T cells during the secondary response has not been evaluated. It is possible that naïve antigen-specific CD4^+^ T cells play a previously unappreciated role in responding to *C*. *trachomatis* following secondary infection. Our goal was to take advantage of antigen-specific NR1 CD4^+^ T cells against *C*. *trachomatis* to tease apart differences between naïve and memory NR1 cells during secondary infection. Understanding the differences between these two populations will help with designing T cell-based vaccines by knowing whether to target a memory T cell population, or both naïve and memory T cell populations. Using RNA sequencing and flow cytometry, we find that memory NR1 T cells are enriched in the genital tract following secondary infection, but that lymph node naïve NR1 T cells are more proliferative. Our data help define differences between these two populations of antigen-specific CD4^+^ T cells in the context of *C*. *trachomatis* infection.

## Results

### RNA sequencing of memory and naïve NR1 T cells shows increased proliferation of naïve NR1 T cells

**Fig 1:**
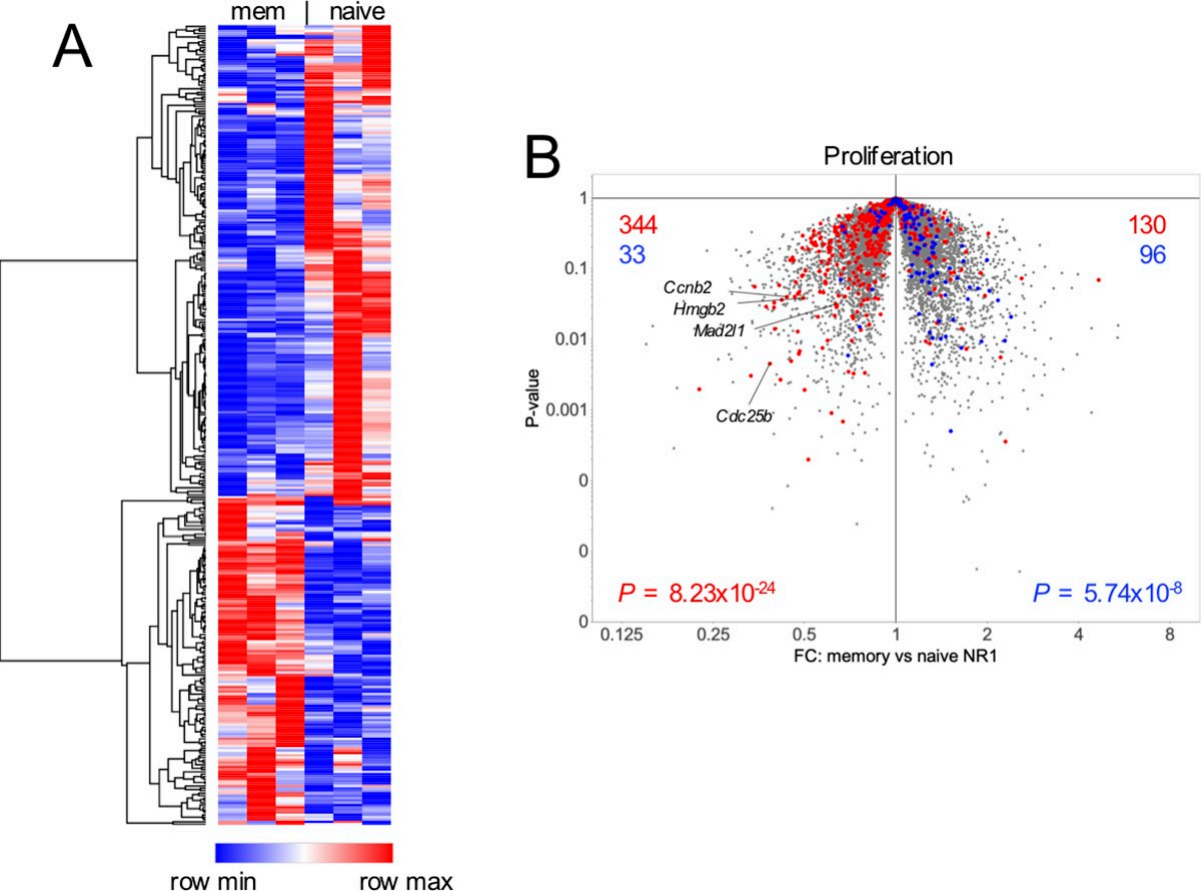
Memory and naïve NR1 T cells have distinct expression profiles. CD90.2^+/+^ mice received 10^6^ CD90.1^+/-^ NR1 T cells one day prior to transcervical infection with 5x10^6^ IFU of *C*. *trachomatis*. Four weeks later, mice received 10^6^ CD90.1^+/+^ NR1 T cells and were subsequently reinfected with 5x10^6^ IFU of *C*. *trachomatis*. Five days post-secondary infection, draining iliac lymph nodes were harvested and equivalent numbers of memory (CD90.1^+/-^) and naïve (CD90.1^+/+^) NR1 T cells were sorted and transcriptomes of the two populations were analyzed by RNA-seq. (A) Heat map of ~350 transcripts twofold differentially expressed. Blue denotes downregulation, red denotes upregulation. (B) Volcano plot of transcriptomes from memory vs naïve NR1 T cells. Genes upregulated in memory T cells are on the right, genes upregulated in naïve T cells are on the left. Proliferation up- (red) or down- (blue) signatures were superimposed from *in vitro*-activated T cells and the total number of genes up- or down-regulated are shown in red or blue, respectively [21]. FC, fold change. Data were analyzed using χ^2^ test and are representative of three technical replicates each pooled from six mice across two independent experiments.

**Fig 2:**
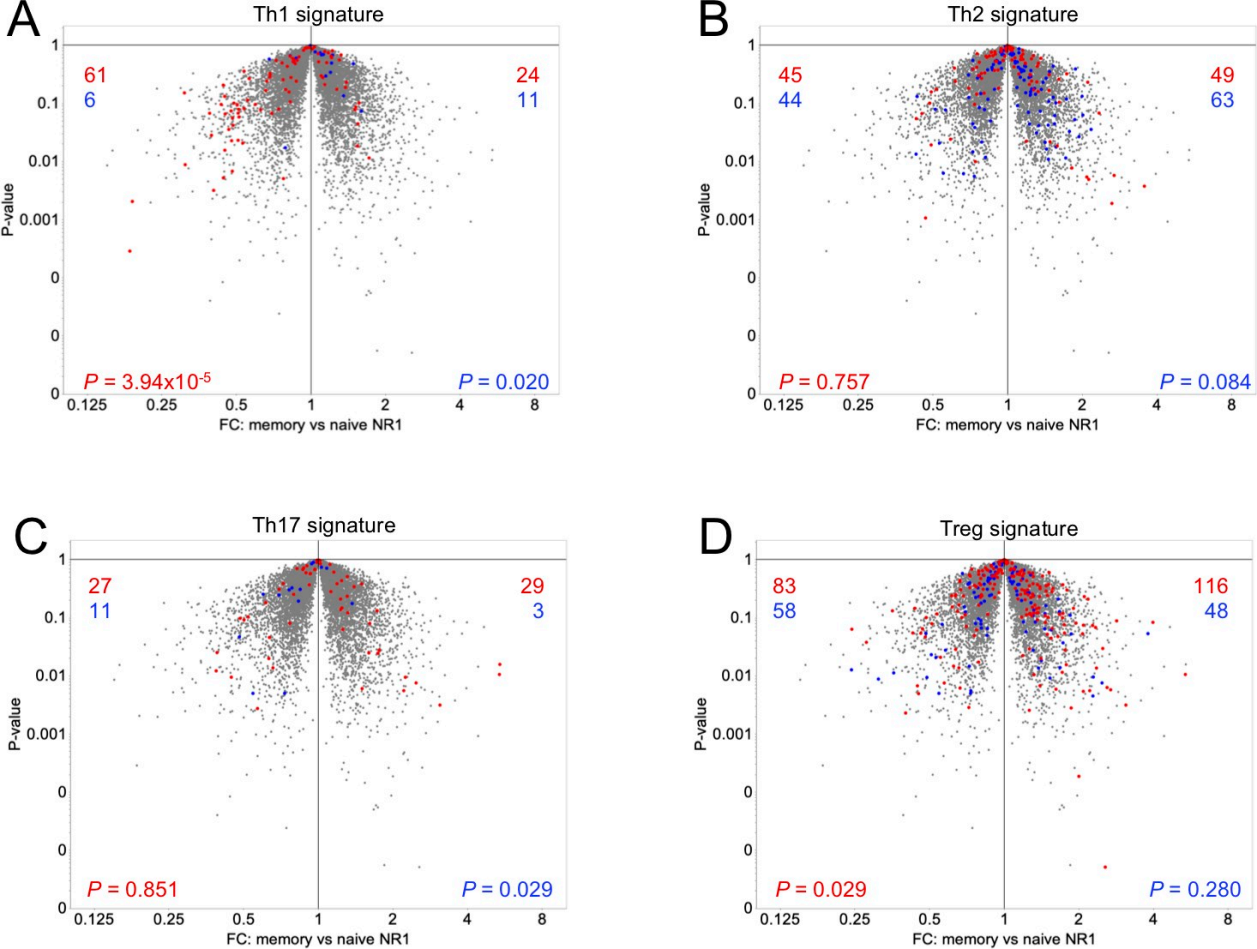
Memory and naïve NR1 T cells exhibit similar T cell signatures. Volcano plots of RNA-seq data comparing genes expressed in memory (right) vs naïve (left) NR1 T cells were superimposed with signatures from (A) *in vitro* Th1 T cells (up–red, down–blue), (B) *in vitro* Th2 T cells (up–red, down–blue), (C) *in vitro* Th17 T cells (up–red, down–blue), and (D) regulatory T cells (Tregs, up–red, down–blue) [21, 22]. FC, fold change. Data were analyzed using χ^2^ test and are representative of three technical replicates each pooled from six mice across two independent experiments.

Phenotypic differences in memory versus naïve antigen-specific CD4^+^ T cells in response to *C*. *trachomatis* infection have never been simultaneously examined in one animal. To study this, we developed a cell transfer-based approach to identify differences between memory and naïve CD4^+^ T cells specific for *C*. *trachomatis* following secondary infection. Naïve B6 mice (CD90.2^+/+^) received CD90.1^+/-^ NR1 T cells one day prior to transcervical infection with *C*. *trachomatis*. Infected mice were allowed to rest for four weeks, allowing the CD90.1^+/-^ NR1 T cells to clear the infection and establish a memory population. These mice then received CD90.1^+/+^ T cells and were reinfected with *C*. *trachomatis* one day later. Five days post-secondary infection, mice were sacrificed and draining iliac lymph nodes harvested and processed for flow cytometry. Equivalent numbers of memory (CD90.1^+/-^) and naïve (CD90.1^+/+^) NR1 T cells were double sorted and subjected to RNA sequencing (RNA-seq) to analyze the transcriptomes of these two different populations. We found subtle but distinct differences in the two populations, and identified ~350 genes that were twofold or more differentially expressed (Fig 1A). Gene-set enrichment analysis (GSEA) revealed upregulation of cell cycle genes in naïve NR1 T cells. We found naïve NR1 T cells had significantly upregulated proliferation transcripts (including *Ccnb2*, *Hmgb2*, *Mad2l1*, *Cdc25b*) compared to memory NR1 T cells (Fig 1B). Naïve NR1 T cells also expressed slightly more genes associated with Th1 cells relative to memory NR1 T cells (Fig 2A), but there was no difference in transcripts associated with Th2, Th17 or Treg phenotypes (Fig 2B–2D). The subtlety of the differences in gene expression we observed may be because both NR1 populations are homogenous T cell populations that contain a pre-arranged T cell receptor. As such, differences in gene expression might have been greater if NR1 cells were compared to endogenous naïve or memory CD4^+^ T cells.

### Memory NR1 T cells home to the genital tract and are activated faster than naïve NR1 T cells

**Fig 3:**
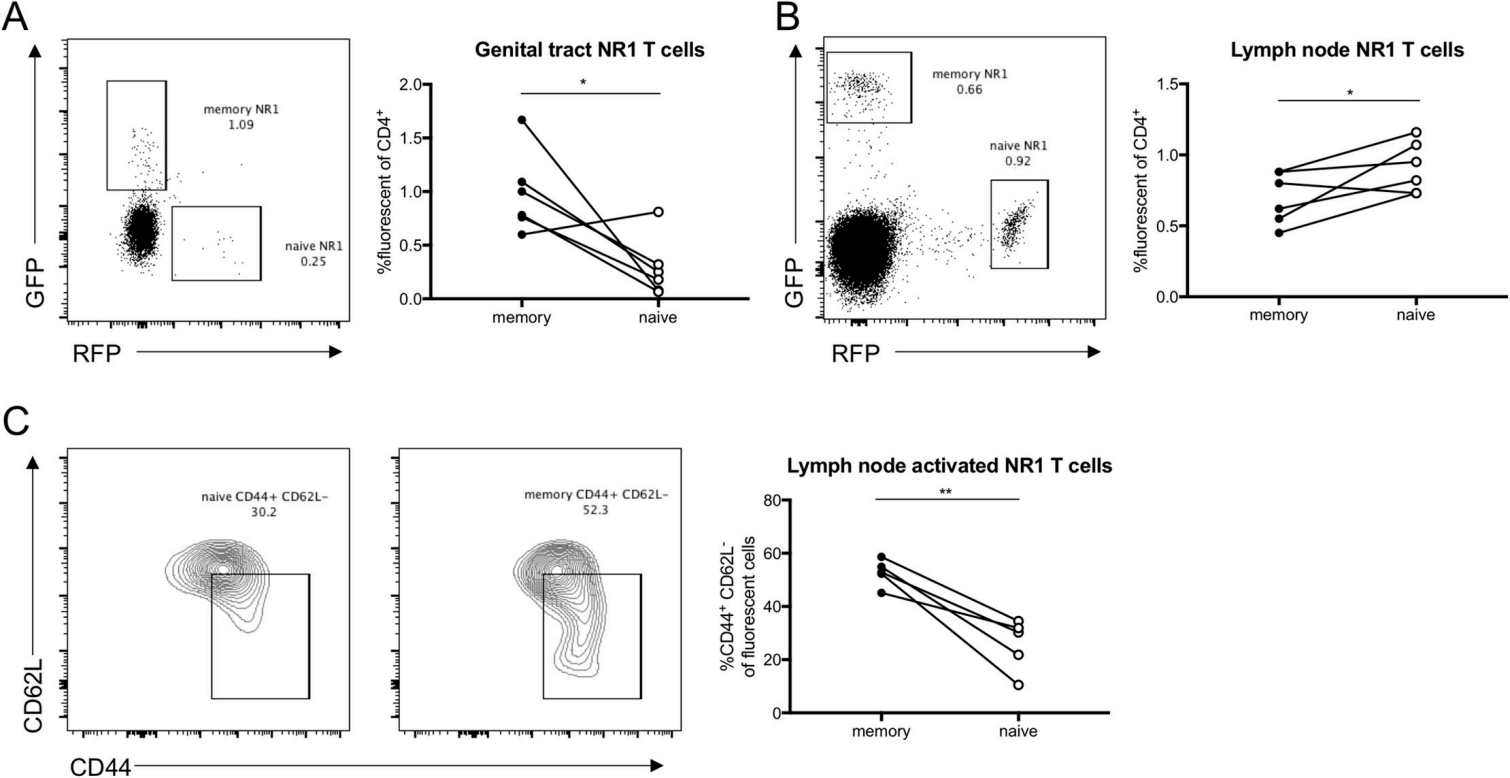
Memory NR1 T cells traffic to the genital tract faster and are more activated than naïve NR1 T cells. Mice received 10^6^ GFP NR1 T cells one day prior to transcervical infection with 5x10^6^ IFU of *C*. *trachomatis*. Four weeks later, mice received 10^6^ RFP NR1 T cells and were subsequently reinfected with 5x10^6^ IFU of *C*. *trachomatis*. Five days post-secondary infection genital tracts were assessed by flow cytometry for (A) NR1 T cells. Draining lymph nodes were also assessed for (B) NR1 T cells and (C) activated NR1 T cells. Data were analyzed using paired *t* test and are representative of at least two independent experiments with five mice each.

We next sought to compare the abundance of the two different NR1 T cell populations in the genital tract and draining lymph nodes. Using a similar approach as described previously, we transferred GFP^+^ NR1 T cells into mice prior to infection and allowed them to rest for four weeks to establish a memory population. We then transferred RFP^+^ NR1 T cells into mice prior to secondary infection to compare memory (GFP^+^) and naïve (RFP^+^) NR1 T cells by flow cytometry. We observed significantly more memory NR1 T cells (GFP^+^) compared to naïve NR1 T cells (RFP^+^) in the genital tract (Fig 3A). In the lymph nodes, there were more naïve NR1 T cells than memory NR1 T cells (Fig 3B). Given that naïve NR1 T cells are more proliferative than memory NR1 T cells, the increased number of naïve NR1 T cells in the lymph node was consistent with our RNA-seq data. We did find that lymph node memory NR1 T cells were significantly more activated, as measured by CD44^+^ CD62L^-^ expression (Fig 3C). Our data suggest that following secondary infection, memory NR1 T cells are able to respond to antigen faster than naïve NR1 T cells, as they are more activated and traffic to the genital tract faster than naïve NR1 T cells.

**Fig 4:**
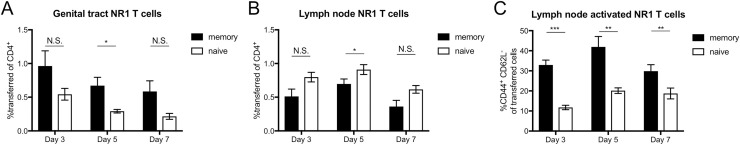
Memory NR1 T cells are more activated than naïve NR1 T cells at multiple time points. Mice received 10^6^ RFP NR1 T cells one day prior to transcervical infection with 5x10^6^ IFU of *C*. *trachomatis*. Four weeks later, mice received 10^6^ GFP NR1 T cells and were subsequently reinfected with 5x10^6^ IFU of *C*. *trachomatis*. Three, five and seven days post-secondary infection genital tracts were assessed by flow cytometry for (A) NR1 T cells. Draining lymph nodes were also assessed for (B) NR1 T cells and (C) activated NR1 T cells. Data were analyzed using paired *t* test and are representative of one experiment with 5 mice per group.

We next assessed these populations at different time points to determine the kinetics of the T cell response following secondary infection. We transferred NR1 T cells prior to primary and secondary infection and quantified memory and naïve NR1 T cells at three, five, and seven days post-secondary infection. In the genital tract, we again observed significantly more memory NR1 T cells at day five post-secondary infection. While not significant at days three and seven, there were more memory NR1 T cells at these time points as well (Fig 4A). In the draining lymph node, we again observed trends towards more naïve NR1 T cells at day three and seven (Fig 4B). At all time points, memory NR1 T cells were significantly more activated than naïve NR1 T cells (Fig 4C). Taken together, our data suggest that antigen experienced memory NR1 T cells are activated rapidly and traffic to the genital tract in greater numbers than naïve NR1 T cells following secondary infection.

### Memory NR1 T cells do not influence the naïve NR1 T cell population

**Fig 5:**
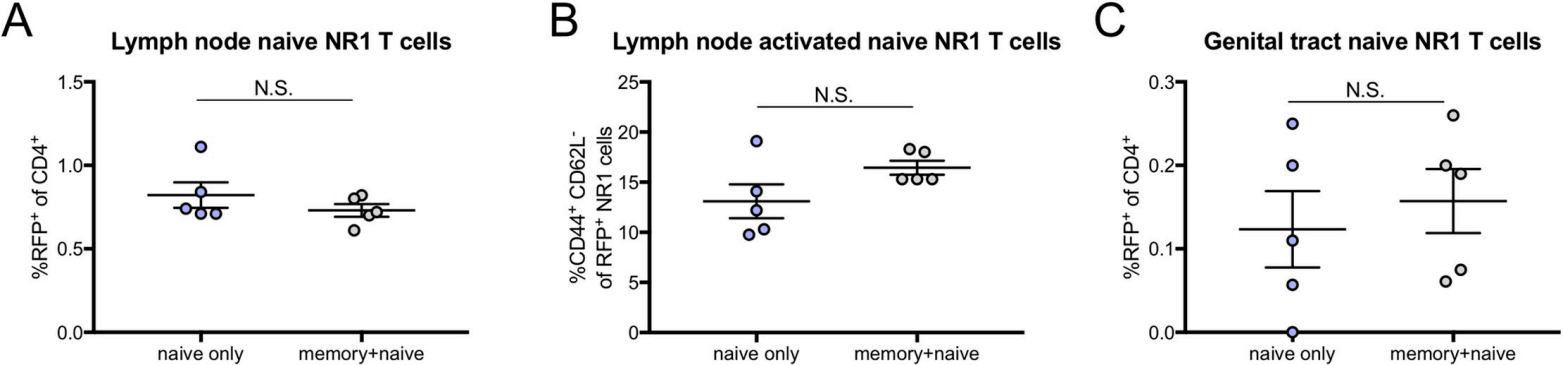
Memory NR1 T cells do not influence the naïve NR1 T cell population. “Naïve” mice were transcervically infected with 5x10^6^ IFU *C*. *trachomatis* and “memory + naïve” mice received 10^6^ GFP NR1 T cells one day prior to infection. Four weeks later, both groups of mice received 10^6^ RFP NR1 T cells and were subsequently reinfected with 5 x 10^6^ IFU *C*. *trachomatis*. Five days post-secondary infection, draining lymph nodes were assessed by flow cytometry for (A) naïve NR1 T cells and (B) activated naïve NR1 T cells. (C) Genital tracts were also assessed for naïve NR1 T cells by flow cytometry. All data were analyzed using unpaired *t* test and are representative of one experiment with five mice per group.

To ensure that the pre-existing memory NR1 T cell population does not play a role in accumulation and activation of naïve NR1 T cells, we compared naïve NR1 T cell populations in mice that either received both memory and naïve NR1 T cells or only naïve NR1 T cells. In the draining lymph node, there were no differences in total (Fig 5A) or activated (Fig 5B) naïve NR1 T cells in mice that received only naïve NR1 T cells versus those that received both memory and naïve NR1 T cells. Furthermore, there were no differences in total naïve NR1 T cells in the genital tract in mice that received only naïve NR1 T cells or both memory and naïve NR1 T cells (Fig 5C). Together, these data suggest that the pre-existing memory NR1 T cell population does not influence the naïve NR1 T cell population.

## Discussion

It has been well established that antigen-specific CD4^+^ T cells are necessary and sufficient for clearing *C*. *trachomatis* infection in mice [6, 7, 23] and transgenic NR1 T cells specific for the *C*. *trachomatis* protein Cta1 [17] can be used to track this response. While naïve NR1 T cells can traffic to the genital tract following *C*. *trachomatis* infection [6, 9, 17], memory NR1 T cells are critical for protection against secondary *C*. *trachomatis* infection [24]. Here, we compared differences between naïve and memory antigen-specific NR1 T cells in the same mouse to discover the relative contributions of these two cell types following secondary infection.

Using RNA-seq, we found that lymph node naïve NR1 T cells are more proliferative than memory NR1 T cells, but that memory NR1 T cells are enriched in the genital tract (Figs 1 and 3). This is contrary to several other reports that found memory T cells were more proliferative than naïve T cells because they required less antigen to become activated and expand [25–28]. While we do observe enhanced memory NR1 T cell activation in the lymph node (Fig 3), enhanced naïve NR1 T cell proliferation might depend on the timepoint and anatomical site analyzed. It is possible that by day five post-secondary infection, memory NR1 T cells that remain in the lymph node are simply the remnants of an expanded population that is now found in the genital tract. If we conducted RNA-seq analysis at an earlier time point in the lymph node, perhaps more consistent with memory NR1 proliferation, or sorted memory and naïve NR1 T cell populations from the genital tract, it is possible that memory NR1 T cells would exhibit more proliferative characteristics. Similarly, we could assess Ki67 expression (a marker associated with cell cycle entry and cellular proliferation) in the genital tract at different time points to determine if genital tract memory T cells are more proliferative than naïve T cells.

It is also important to note that the memory NR1 T cells we observed in the genital tract by flow cytometry could be tissue resident memory T cells (Trms) rather than cells that had expanded in the lymph node during secondary infection. Tissue resident memory T cells differ from other memory T cells as they remain in peripheral tissues even after infection has been cleared and do not recirculate through the lymphatic tissue [29, 30]. It has been shown that Trms can form following *C*. *trachomatis* infection, and protection against secondary infection can be partially attributed to these cells [24]. As such, it is possible that the memory T cell population we observe in the genital tract could be Trms rather than memory NR1 T cells that have trafficked to the genital tract from the draining lymph node. Determining the identity of the memory NR1 T cells in the genital tract will be the topic of future studies and will help elucidate further differences between memory and naïve NR1 T cells.

While there are many questions still to be answered, our work does highlight broad differences in memory and naïve antigen-specific CD4^+^ T cells during secondary *C*. *trachomatis* infection in mice. The data presented here can be used as a platform for many future studies, including investigating the mechanism of antigen-specific memory CD4^+^ T cell mediated protection against *C*. *trachomatis*, characterizing differences between naïve and memory antigen-specific CD8^+^ T cells, or even broadly comparing both antigen-specific CD4^+^ and CD8^+^ T cells. This work will help build a better understanding of how T cells mediate protection against *C*. *trachomatis* and further the ultimate goal of developing an effective T cell vaccine against *C*. *trachomatis*.

## Conclusions

There are two major limitations to this study. First, RNA-seq revealed distinct transcriptional profiles between memory and naïve NR1 T cells following *C*. *trachomatis* infection. While RNA-seq is certainly a valuable tool for identifying differences in cell populations, validation experiments are necessary to confirm these findings. We were able to identify enhanced naïve NR1 T cells in the lymph nodes of mice, suggesting that naïve NR1 T cells do proliferate more than memory NR1 T cells. However, testing of cell proliferation through dye incorporation or proliferation markers is necessary to confirm these findings. Furthermore, transcription factor staining of memory and naïve NR1 T cells is necessary to confirm the T cell subsets we observed through RNA-seq. Second, while we strove to detect differences in naïve and memory NR1 T cells following *C*. *trachomatis* infection, our naïve T cell population can actually be more accurately described as early effector CD4^+^ T cells, as this population has encountered antigen and begun to proliferate. Our terminology of naïve and memory reflects the status of these T cells at the point of secondary infection, but to more accurately identify differences in these two populations, we will need to conduct further experiments comparing memory NR1 T cells to naïve NR1 T cells in mice that have not received secondary infection. These experiments are the topic of future studies, and our work here provides a comprehensive foundation in studying two distinct populations of antigen specific CD4^+^ T cells in a mouse model of *C*. *trachomatis* infection.

## Materials and methods

### Growth and isolation of bacteria

*Chlamydia trachomatis* serovar L2 (434/Bu; ATCC) was propagated in McCoy cells as previously described [18, 31]. Aliquots of purified elementary bodies were stored at -80°C in medium containing 250 mM sucrose, 10 mM sodium phosphate and 5 mM L-glutamic acid (SPG buffer) and thawed immediately prior to use.

### Mice

Six- to eight-week old female C57BL/6 mice, C57BL/6-Tg(CAG-EGFP)1Osb/J (β-actin GFP), and B6.Cg-Tg(CAG-mRFP1)1F1Hadj/J (β-actin RFP) were purchased from The Jackson Laboratory (Bar Harbor, ME). DPE-GFP mice were described as previously [32]. TCR transgenic OT-II mice that recognize OVA_323-339_ were purchased from The Jackson Laboratory and backcrossed onto the CD45.1 background (by Dr. U. von Andrian). NR1 mice (CD90.1^+/+^ or CD90.1^+/-^ background) that recognize the *C*. *trachomatis* antigen Cta1_133-152_ have been described previously [17]. To generate GFP NR1 or RFP NR1 mice, GFP or RFP and NR1 mice were crossed and assessed by flow cytometry for GFP or RFP expression. All mice were housed in the Harvard Medical School Center for Animal Resources and Comparative Medicine in specific pathogen free conditions in standard ventilated cages (Techniplast), with a cycle of 14 hours light/10 hours dark. All experiments were approved by Harvard’s Institutional Animal Care and Use Committee. All mice were sacrificed using carbon dioxide followed by cervical dislocation according to AVMA guidelines for the euthanasia of animals.

### T cell adoptive transfers and infection of mice

Mice were treated with 2.5 mg medroxyprogesterone subcutaneously one week prior to infection. CD4^+^ T cells were isolated from secondary lymphoid organs of transgenic mice and recipient mice were injected with 10^6^ naïve T cells intravenously one day prior to infection. Mice were infected transcervically with 5x10^6^ inclusion forming units (IFU) of *C*. *trachomatis* in 10 μl SPG buffer using an NSET pipet tip (ParaTechs, Lexington, KY) as described previously [6]. Mice were then treated with another round of 2.5 mg medroxyprogesterone three weeks following primary infection. Six days later, 10^6^ naïve transgenic T cells were transferred intravenously into recipient mice and mice were infected transcervically the following day. At three, five or seven days post-secondary infection, mice were sacrificed and the upper genital tract (uterine horns and ovaries) and iliac lymph nodes were harvested. For all experiments except RNA sequencing, 5 mice per group were used. For RNA sequencing, 6 mice were pooled for each of 3 technical replicates. Mice were monitored weekly to identify any animals in undue stress or pain that needed to be euthanized immediately, as determined by ruffled fur and limited movement. However, no mice in this study needed to be euthanized early as there were no animals exhibiting undue stress or pain. Time points were chosen to maximize the numbers of T cells in the genital tract and draining lymph nodes following secondary *C*. *trachomatis* infection.

### Preparation of tissue

Single-cell suspensions of secondary lymphoid organs were prepared by grinding the tissue between frosted microscope slides. Uteri were minced with scalpels and enzymatically dissociated in HBSS/Ca2^+^/Mg2^+^ containing 1 mg/ml type XI collagenase and 50 Kunitz/ml DNase for 30 min at 37˚C, washed in Ca2^+^/Mg2^+^-free PBS containing 5 mM EDTA, and then ground between frosted microscope slides prior to filtration through a 70-μm mesh.

### Flow cytometry

All antibodies were purchased from BioLegend and were used at a dilution of 1:400 except where noted. Cells were stained with surface and activation markers along with anti-FcRγ (Bio X Cell, 1:100 dilution) following isolation. Cells were incubated with fluorochrome conjugated antibodies against mouse CD3 (clone 17A2, 1:100 dilution), CD4 (clone GK1.5) or CD4 (clone RM4-5; Invitrogen), CD90.1 (OX-7), CD90.2 (clone 53–2.1), CD8 (clone 53–6.7), CD44 (clone 1M7), CD62L (clone Mel-14) and a live/dead fixable aqua dead cell stain kit to exclude dead cells (1:100 dilution, Invitrogen). Data were collected on an LSR II (BD Biosciences) and analyzed using FlowJo (Tree Star, Ashland, OR).

### RNA sequencing and transcriptome analysis

Cells were sorted and processed according to the Immgen protocol for ultra-low-input RNA-seq analysis as described previously [33, 34]. Briefly, iliac lymph nodes from six individual mice were pooled per biological replicate. RNA sequencing was conducted on biological triplicates of equal numbers (no more than 10^3^ cells) of memory and naïve NR1 T cells that were double-sorted by FACSAria into 5 μl TCL buffer (Qiagen) containing 1% 2-Mercaptoethanol (Sigma). Smart-Seq2 library preparation and sequencing were completed as described previously [33, 35]. Transcript quantification was performed using the Broad Technology Labs computational pipeline with Cuffquant version 2.2.1 [36]. Reads were further filtered by minimal expression in Multiplot Studio and analyzed using Multiplot Studio (GenePattern, Broad Institute). GSEA was performed using GSEA 4.0.1 and the gene sets database Hallmark version 7.0. A heatmap showing differentially expressed genes was generated using Morpheus software (Broad Institute). The sequencing data are available in the Gene Expression Omnibus under data repository accession no. GSE146265.

### Statistical analysis

Volcano plot enrichment *P* values were determined using χ^2^ test in Microsoft Excel. Statistical analysis for flow cytometry data was performed using Prism (GraphPad). Differences were considered statistically significant if the *P* value was less than 0.05. *P < 0.05, **P < 0.01, *** *P* < 0.001; ****P < 0.0001. Except where noted, data are represented as mean ± SEM.

## Supporting information

S1 File The ARRIVE guidelines 2.0: Author checklist. (PDF)
